# Direct visualization-guided PENG block (Pericapsular nerve Group): optimizing postoperative analgesia in total hip arthroplasty

**DOI:** 10.1186/s13018-026-06676-9

**Published:** 2026-02-07

**Authors:** Maria Bautista, Jacobo Triviño-Arias, María Camila Gómez-Ayala, Alejandro Gallego, Andrés Bonnet, Alfredo Sánchez-Vergel

**Affiliations:** 1 Servicio de Ortopedia y Traumatología, Fundación Valle del Lili, Valle del Cauca, Cali, Colombia; 2 Facultad de Ciencias de la Salud, Universidad ICESI, Valle del Cauca, Cali, Colombia; 3 Centro de Investigaciones Clínicas, Fundación Valle del Lili, Valle del Cauca, Cali, Colombia; 4 Departamento de Anestesiología, Fundación Valle del Lili, Valle del Cauca, Cali, Colombia

**Keywords:** Total hip arthroplasty, Direct anterior approach, Regional analgesia, PENG block, Postoperative pain

## Abstract

**Background:**

The pericapsular nerve group (PENG) block has proven to be an effective strategy for postoperative pain management in total hip arthroplasty (THA). However, its implementation requires specialized equipment and expertise, which limits its reproducibility. The objective of this study was to describe the technique of direct visualization-guided PENG block (PENG-DV) and assess its outcomes for postoperative pain control.

**Methods:**

A retrospective cohort study was conducted in patients who underwent THA through a direct anterior approach (DAA) and received the PENG-DV block as part of a standardized multimodal analgesia protocol. Demographic variables, pain score on the visual analogue scale (VAS), opioid consumption, muscle strength, and ambulation within the first 24 h were evaluated.

**Results:**

Of the 128 patients identified, 112 were included in the analysis. The cohort was predominantly female (60.7%), with a mean age of 63 years. The median postoperative VAS score was 2 and 3 at 12 and 24 h respectively, increasing to 5 during physical therapy. By postoperative day one, 83.9% of patients ambulated, and most achieved muscle strength ≥ 3 for both hip flexion and knee extension. A total of 54.5% of patients required opioid rescue, with a mean morphine equivalent consumption of 10 mg within the first 24 h.

**Conclusion:**

PENG-DV for THA via DAA appears to be a promising strategy for effective pain control in the immediate postoperative period, as part of a multimodal analgesic approach designed to reduce opioid consumption and promote early mobilization.

## Background

Total hip arthroplasty (THA) has consistently demonstrated excellent long-term outcomes [1]; however, postoperative pain management remains a challenge in the early postoperative period. Effective pain control facilitates early mobilization and prevent postoperative complications [2, 3]. Furthermore, optimization of postoperative multimodal analgesia reduces opioid consumption, minimizes adverse effects and associated costs [4–6], and promotes the implementation of fast-track clinical pathways and outpatient surgery [7].

The pericapsular nerve group (PENG) block, first described by Girón-Arango et al., is an effective technique for controlling pain, reducing opioid requirements, and facilitating early rehabilitation in patients with hip fractures [7]. In primary THA, the use of PENG block resulted in statistically significant reductions in pain scores compared with placebo [8–10]. However, the conventional PENG block technique requires trained anesthesiologists and the use of ultrasound guidance or nerve stimulators, which limits its broad implementation.

The direct anterior approach for THA allows direct visualization of both the anterior capsule and the anatomical landmarks used for the PENG block. Cadaveric studies have shown that infiltration of the pericapsular branches of the femoral and accessory obturator nerves can be effectively achieved through this approach, with a distribution comparable to that achieved by the ultrasound-guided PENG block [11–13].

The aim of this study is to describe the pericapsular nerve group block under direct visualization (PENG-DV) performed during primary THA via the direct anterior approach, and to assess its impact on postoperative pain control.

## Methods

A retrospective observational cohort study was conducted, including adult patients with primary or secondary osteoarthritis who underwent primary THA via the direct anterior approach under spinal anesthesia, in whom the PENG-DV block was performed between January 2023 and January 2025. Patients with physical or cognitive impairments limiting the applicability of pain scales, those requiring conversion from spinal to general anesthesia, and patients with contraindications to opioid use or allergies to contrast media or local anesthetics were excluded.

According to the institutional protocol, all patients received a standardized analgesic regimen consisting of 1 g of oral acetaminophen before entering the operating room, 4 mg of intravenous dexamethasone at the start of surgery, and 4 mg of ondansetron at the end of the procedure. Postoperative pain management in the recovery unit and during hospitalization included of acetaminophen 1 g every 8 h, celecoxib 200 mg every 12 h, dipyrone 1250 mg every 4 h, and opioid rescue analgesia of hydromorphone 0.4 mg every 4 h as needed.

A retrospective review of medical records was performed to collect demographic data, VAS pain scores at 12 and 24 h, opioid rescue requirements within the first 24 h, ability to ambulate with a walker and lower limb strength on postoperative day one, and the presence of lateral femoral cutaneous nerve sensory deficit at the 15-day postoperative follow-up.

### Statistical analysis

Continuous data were analyzed according to distribution (Shapiro–Wilk test). Variables with normal distribution were expressed as mean and standard deviation, while non-normally distributed data were reported as median and interquartile range. Categorical variables were summarized as proportions. Statistical analyses were performed using STATA version 17.0 (StataCorp LLC, TX, USA).

### Surgical technique

The anterior capsule of the hip is mainly innervated by the obturator nerve, the accessory obturator nerve, and the femoral nerve, whose articular branches emerge near the iliopectineal eminence and the anterior inferior iliac spine [14]. The direct anterior approach for THA provides clear visualization of these anatomical landmarks, allowing for the selective administration of local anesthetics.

**Fig. 1:**
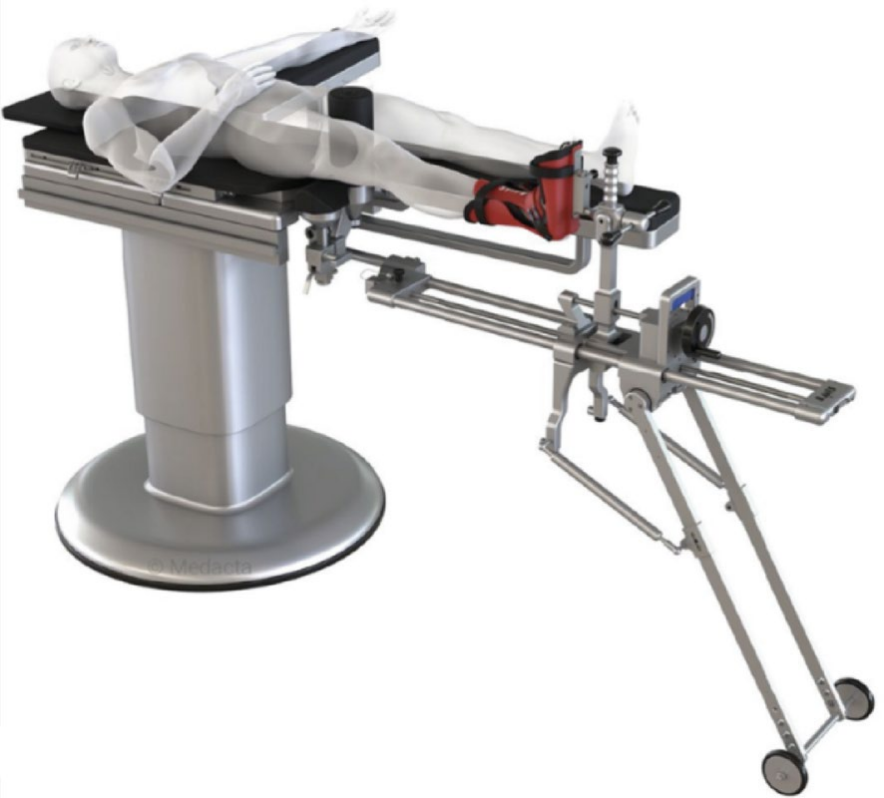
AMIS mobile leg positioner© (16)

**Fig. 2:**
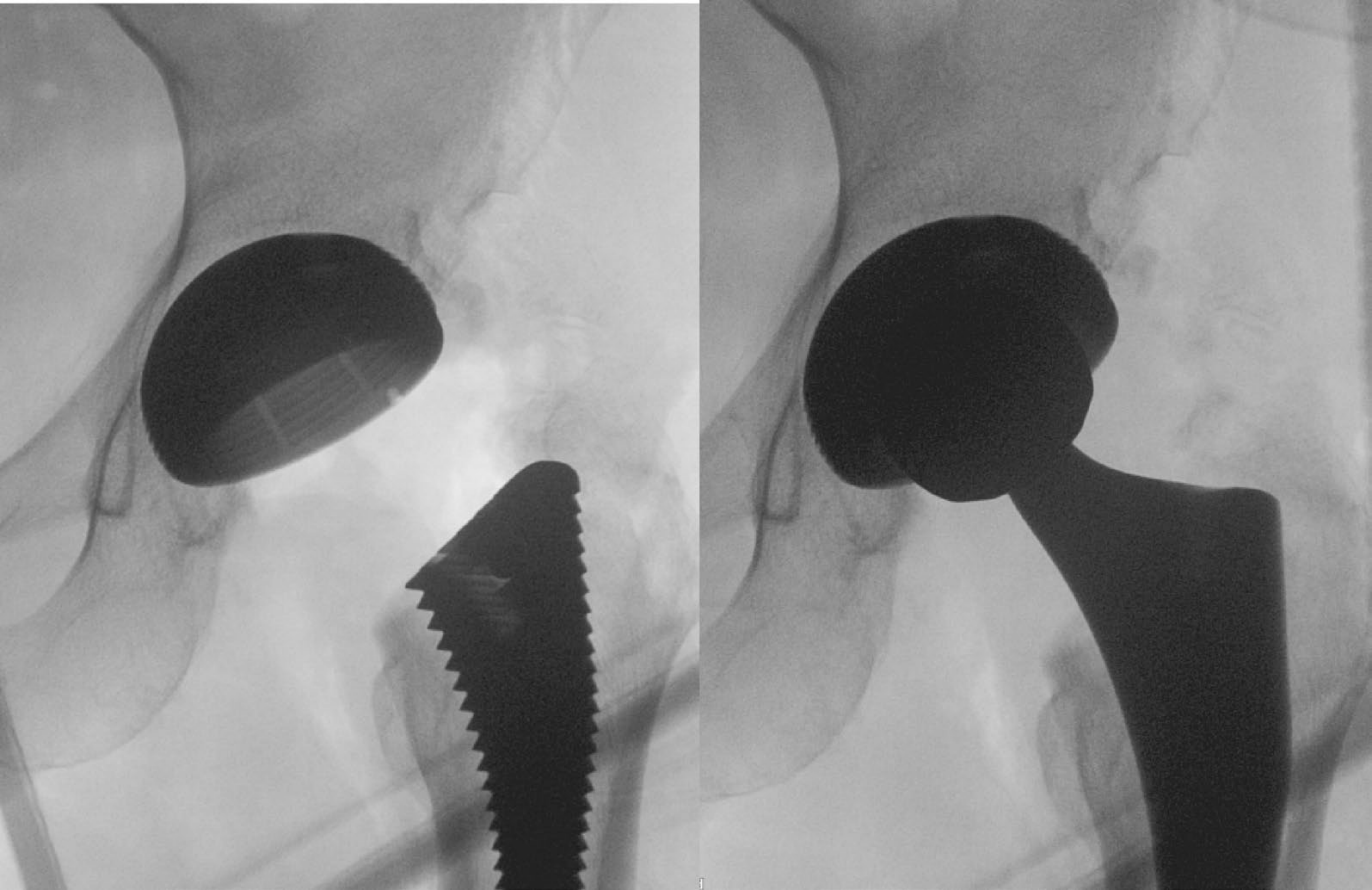
Fluoroscopic visualization of the final acetabular cup

**Fig. 3:**
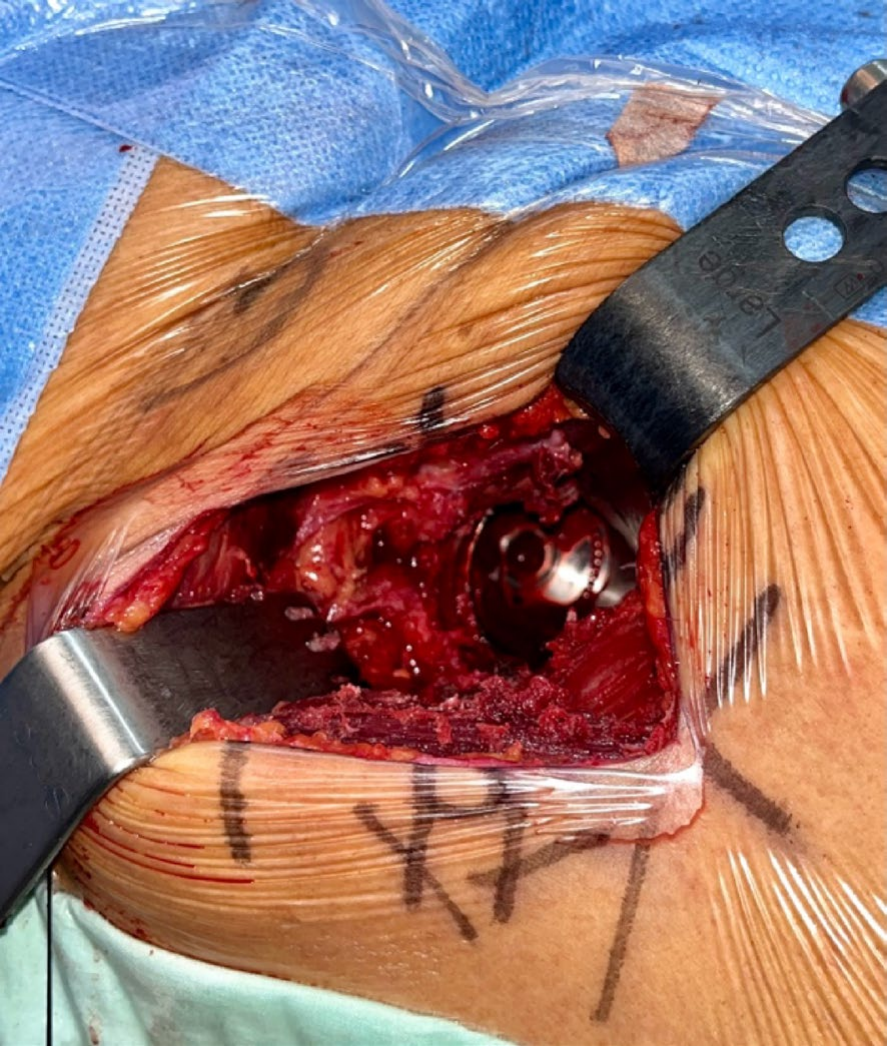
Direct visualization of the definitive acetabular component

To describe the PENG-DV block, the minimally invasive anterior approach (AMIS) for THA is detailed [15]: patients were positioned supine on the operating table, with the operative leg secured in the AMIS Mobile Leg Positioner© (Fig. 1) [16]. A 6 to 12 cm incision was made lateral to the anterior superior iliac spine and extended toward the fibular head. The intermuscular plane between the tensor fasciae latae and sartorius was developed, then the lateral circumflex vessels were identified and ligated, and the joint capsule exposed. A capsulotomy was performed to visualize the femoral neck osteotomy was performed as planned [14, 15]. After the femoral head was extracted, the pubofemoral ligament and the superior and lateral portions of the capsule were released. To expose the proximal femur, the limb was placed in maximal external rotation and extension, then it was prepared with sequential rasps until adequate fit was achieved. With the hip in neutral position, the acetabulum was exposed, the labrum resected, and sequential reaming performed. Stability and leg length were assessed with trial components; definitive implants were placed once satisfactory stability and length were achieved. Correct acetabular cup positioning was confirmed with fluoroscopy and direct visualization (Figs. 2 and 3).

**Fig. 4:**
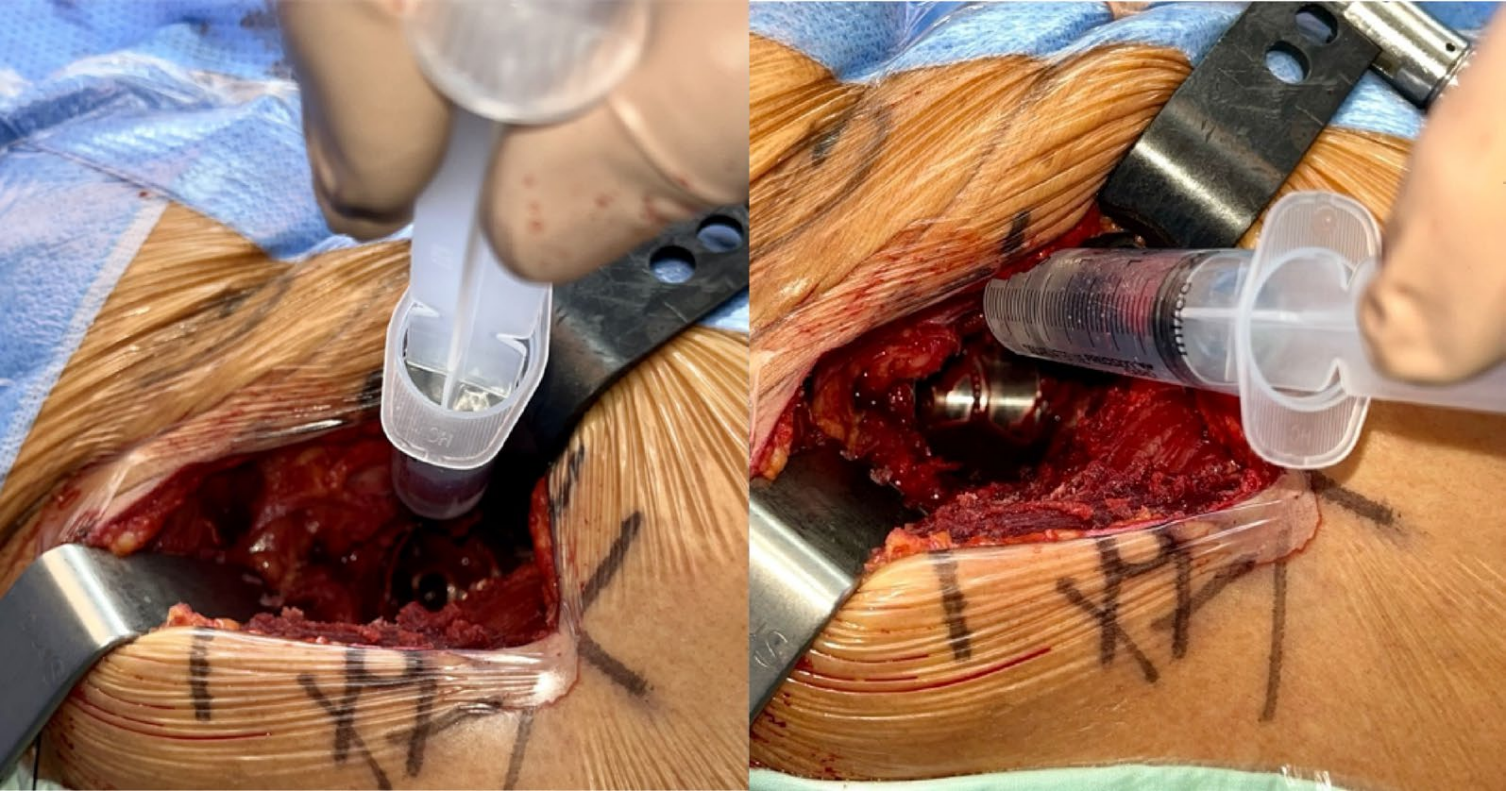
Needle direction and angulation for PENG-DV infiltration

**Fig. 5:**
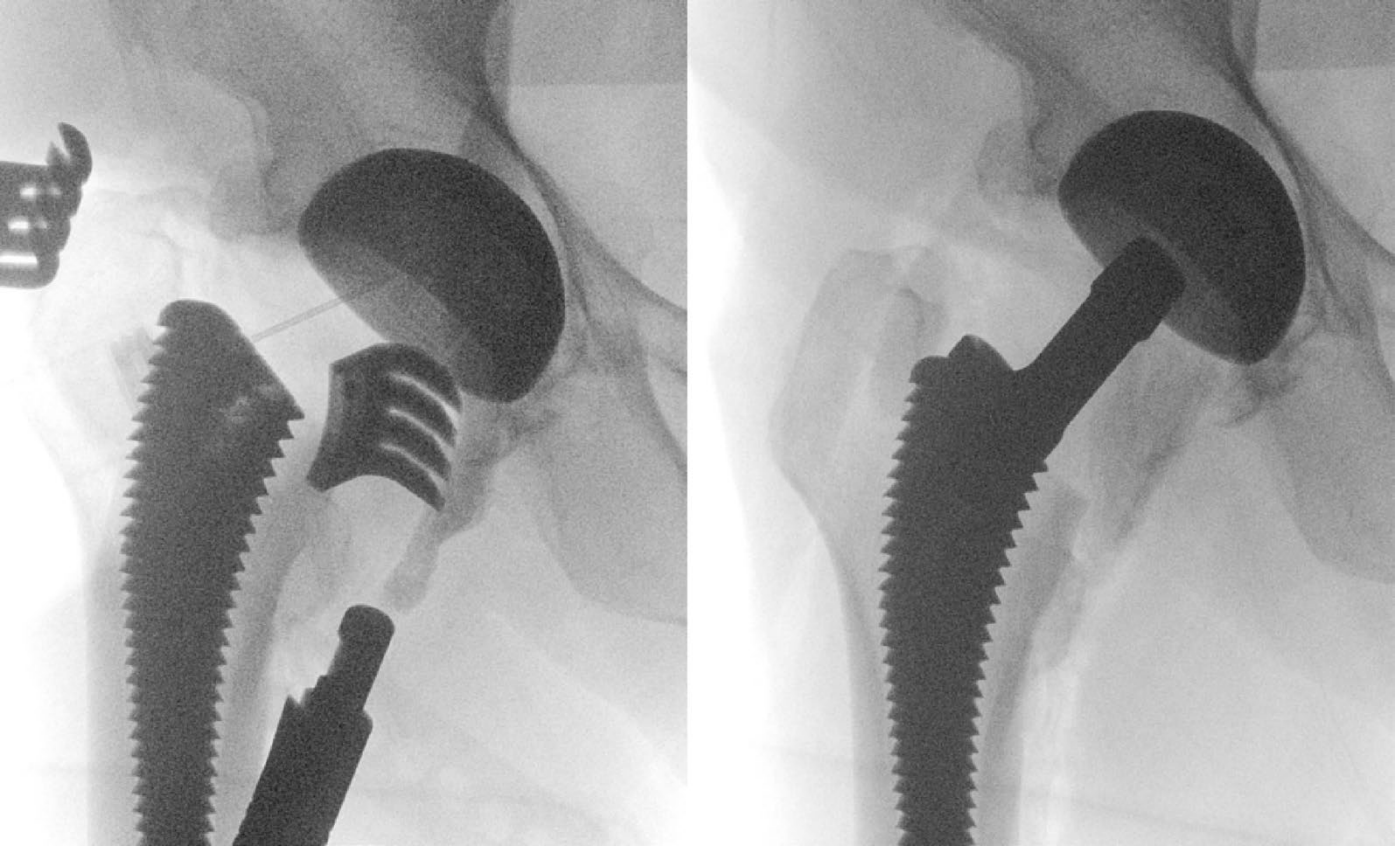
Fluoroscopic visualization of the distribution of the anesthetic–contrast mixture

The PENG-DV block starts with an adequate exposure of the anterior hip capsule following the dissection of the intermuscular plane. After implantation of the definitive acetabular component, the anterior inferior iliac spine (AIIS) and iliopectineal eminence were palpated. A 22-G needle was introduced into the space between the AIIS and the iliopectineal eminence, at an angle of 30–45° relative to the horizontal pelvic plane, advancing from lateral to medial (Fig. 4). The needle was maintained in contact with the bony surface of the iliopectineal eminence to avoid injury of major neurovascular structures. Once correctly positioned, a combination 10 mL of 0.75% bupivacaine, 1 mL of ketorolac (30 mg/mL), 1 mL of epinephrine, and 50 mL of saline was injected. For this study, 10 mL of contrast medium was incorporated into the analgesic solution to allow fluoroscopic visualization. Proper needle positioning ensured optimal distribution of the solution toward the articular branches of the femoral and accessory obturator nerves. Adequate dispersion of the contrast medium along the anterior hip capsule was verified under fluoroscopic guidance (Fig. 5).

## Results

A total of 128 patients were identified during the study period, and 112 were included in the analysis. Patients were excluded if they initially received general anesthesia or required conversion to general anesthesia following a failed spinal anesthetic. Overall, 60.7% of patients were female, the mean age was 63 years (IQR: 56–71), and the mean BMI was 26.5 (IQR: 23.5–28.7) (Table 1).

**Table 1:** Demographic characteristics of the patients included in the study

*N* = 112	
Variables	Median (IQR)
Age (Years)	63 (56–71)
BMI (kg/m^2^)	26.5 (23.5–28.7)
Variables	Proportion
Sex	–
Female (%)	60.7
Male (%)	39.3

A total of 97.3% of patients received 1 g of acetaminophen prior to surgery. The mean anesthesia and surgical times were 131 min (IQR: 120–150) and 100 min (IQR: 90–115), respectively. Regarding intraoperative complications, five patients sustained a greater trochanter fracture, one patient a calcar fracture, one patient a diaphyseal fracture, and one patient an acetabular fracture.

**Fig. 6:**
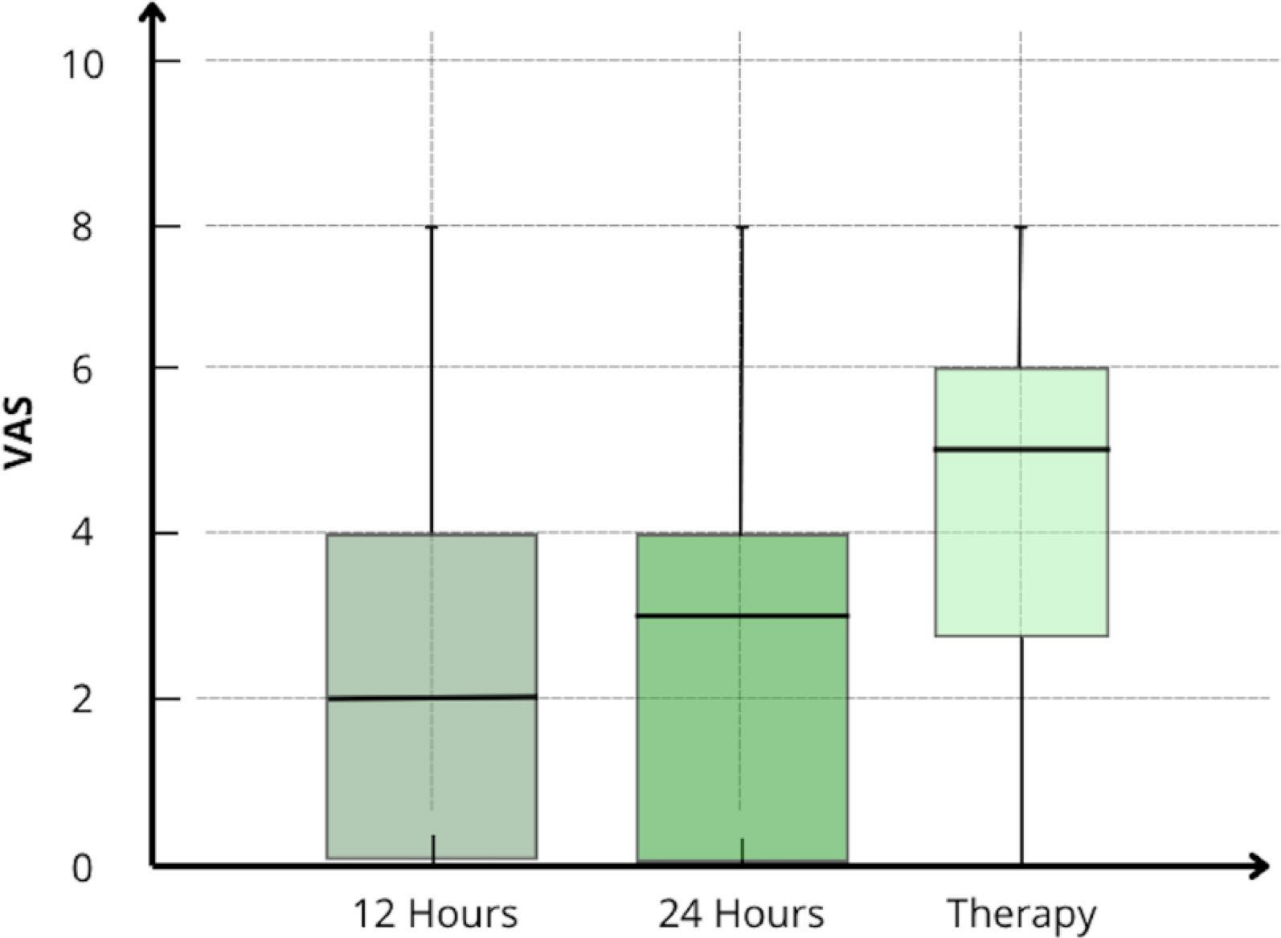
Box plot of median postoperative pain measured at different time points

Median postoperative VAS pain scores were 2 (IQR: 0–4) and 3 (IQR: 0–4) at 12 and 24 h respectively, and 5 (IQR: 3–6) during physical therapy (Fig. 6).

By postoperative day one, 83.93% of patients were able to ambulate with a walker. Median hip flexion strength on the Daniels scale was 4 (IQR: 3–4) at 24 h, with 55.35% achieving strength ≥ 3 for hip flexion and 81.25% for knee extension (Median 4; IQR: 4–4) **(**Table 2**).**

**Table 2:** Muscle strength according to the Daniels scale 24 h post-op

*N* = 112	
Variable	Median (IQR)
Hip flexion strength	4 (3–4)
Knee extension strength	4 (4–4)
Variable	Proportion
Hip flexion strength	
0	0%
1	1.79%
2	42.86%
3	54.46%
4	0.89%
Knee extension strength	
0	0%
1	0%
2	18.75%
3	69.64%
4	11.61%

No cases of femoral nerve motor paralysis or lateral femoral cutaneous nerve sensory deficit were observed at the first postoperative follow-up (15 days).

Opioid rescue analgesia was required in 54.5% of patients, who received one or more doses of 0.4 mg hydromorphone (or 4 mg morphine equivalents) within the first 24 h. The median cumulative opioid consumption, expressed as intravenous morphine equivalents, was 10 mg (IQR: 8–20).

## Discussion

Patients undergoing joint replacement often experience episodes of severe pain (≥ 7) on the first postoperative day and during mobilization [17], thus, additional pain management strategies are recommended to improve clinical outcomes. Among these, the PENG block has been associated with a lower incidence of motor blockade, sedation, nausea, and catheter-related complications compared to epidural analgesia [18], supporting its safety and effectiveness for postoperative pain management. Furthermore, PENG block has demonstrated proven analgesic efficacy in patients with hip fractures, including both intracapsular and extracapsular fractures. Additionally, multiple studies have reported significantly reduced pain scores in patients receiving PENG block compared with other regional anesthesia techniques or placebo [8–10, 19, 20].

The PENG-DV block performed during total hip arthroplasty via the direct anterior approach offers an innovative and effective alternative for postoperative pain management. In contrast to the conventional PENG block, the PENG-DV block is a simple and reproducible intraoperative technique performed under sterile conditions. This technique avoids additional skin punctures and the need for specialists trained in ultrasound or regional anesthesia, without increasing operative time; the median surgical time in our cohort was 100 min (IQR: 90–115). In addition, it enables precise delivery of the analgesic solution, an effective coverage of the articular branches of the femoral and accessory obturator nerves [12, 13].

In the present analysis, patients who received PENG-DV block had a median VAS score of 2 and 3 at 12 and 24 h postoperatively respectively, reflecting adequate acute pain control. In contrast, previous studies have reported mean pain scores up to 7.2 ± 1.7 [21]. Postoperative pain levels typically increase during mobilization [21–24]; however, in our study, median pain score during physiotherapy was 5 (IQR: 3–6), consistent with “moderate pain” as defined by the World Health Organization criteria [25].

A total of 54.5% of patients required at least one rescue opioid dose within the first 24 h, with a mean consumption of 10 mg in intravenous morphine equivalents. These findings are consistent with previous studies demonstrating that the PENG block provides effective analgesia while reducing opioid requirements and related adverse effects [26, 27]. Similarly, Chung et al. [9] reported a significant reduction in intravenous fentanyl-equivalent opioid consumption among patients receiving the PENG block.

Conversely, Kinder et al. [28], in a retrospective analysis comparing 66 patients who received the PENG block with 70 controls, found no significant differences in opioid consumption between the groups. However, these results should be interpreted with caution, as most of their cases were performed via the posterior approach whereas our cohort underwent THA via the direct anterior approach, which has been associated with reduced postoperative pain and faster functional recovery [28].

Despite being performed at a site distant from the femoral nerve, a residual risk of quadriceps motor impairment remains from inadvertent spread of the analgesic solution toward the nerve [9, 29, 30]. Nevertheless, several studies demonstrated that the PENG block facilitates early ambulation and reduces the incidence of quadriceps motor blockade [10, 27, 30–33]. The median Daniels scale for hip flexion and knee extension at 24 h was 4, indicating mild motor weakness; however, 83.9% of patients were able to ambulate on the first postoperative day. Although this finding may have been influenced by factors other than the block itself, functional assessments were conducted by the physical therapy team, who were blinded to its application.

To the authors’ knowledge, this is the first study to describe the direct visualization–guided PENG (PENG-DV) technique and to report its clinical outcomes in patients undergoing total hip arthroplasty, which represents a relevant strength by providing initial evidence on the feasibility of this approach in clinical practice. Nevertheless, the main limitations of this study include its retrospective design, relatively small sample size, and, most importantly, the absence of a control group, which restricts the ability to draw definitive conclusions regarding the clinical effectiveness of the PENG-DV technique. Additionally, the inclusion of both primary and secondary osteoarthritis without stratified analysis, as well as the potential confounding effect of the direct anterior approach, may have influenced the observed outcomes. These limitations reflect the exploratory nature of this early-phase study, intentionally conducted in accordance with the IDEAL framework to describe and standardize a novel technique [34]; comparative analyses and subgroup stratification are planned for subsequent studies. Despite these limitations, our findings demonstrate a favorable trend toward improved pain control and functional recovery following total hip arthroplasty.

## Conclusion

These findings suggest that PENG-DV block during THA via the direct anterior approach is a safe, simple, and potentially effective technique for postoperative pain control. Analgesia was effective within the first 24 h, with a moderate increase in pain during mobilization and low opioid consumption. The PENG-DV block may represent a valuable component of analgesia, enhancing functional recovery and facilitating early discharge pathways. While these results are promising, further comparative studies are necessary to confirm the block’s broader clinical utility, cost-effectiveness, safety, and reproducibility, and to establish its role as an integral part of postoperative pain management protocols.

## Data Availability

The datasets analysed during the current study.

## Abbreviations

PENG: Pericapsular nerve group
THA: Total hip arthroplasty
PENG-DV: Direct visualization-guided PENG
VAS: Visual analogue scale
AMIS: Minimally invasive anterior approach
AIIS: Anterior inferior iliac spine
CEIB: institutional biomedical research ethics committee

## References

[1] Ang JJM, Onggo JR, Stokes CM, Ambikaipalan A. Comparing direct anterior approach versus posterior approach or lateral approach in total hip arthroplasty: a systematic review and meta-analysis. European J Orthop Surg & Traumat. 2023;33(7):2773. Available from: https://pmc.ncbi.nlm.nih.gov/articles/PMC10504117/10.1007/s00590-023-03528-8 PMC10504117 37010580

[2] Sharma V, Morgan PM, Cheng EY. Factors influencing early rehabilitation after THA: a systematic review. Clin Orthop Relat Res. 2009;467(6):1400–11. Available from: https://pubmed.ncbi.nlm.nih.gov/19277807/10.1007/s11999-009-0750-9 PMC2674177 19277807

[3] Rodriguez S, Shen TS, Lebrun DG, Della Valle AG, Ast MP, Rodriguez JA. Ambulatory total hip arthroplasty: causes for failure to launch and associated risk factors. Bone Jt Open. 2022;3(9):684–91. Available from: https://pubmed.ncbi.nlm.nih.gov/36047458/10.1302/2633-1462.39.BJO-2022-0106.R1 PMC9533240 36047458

[4] Shah R, Kuo YF, Westra J, Lin YL, Raji MA. Opioid use and pain control after total hip and knee arthroplasty in the US, 2014 to 2017. JAMA Netw Open. 2020;3(7):e2011972–e2011972. Available from: https://jamanetwork.com/journals/jamanetworkopen/fullarticle/2768844 10.1001/jamanetworkopen.2020.11972 PMC7641514 32729917

[5] Agrawal Y, Smith RM, Garbuz DS, Masri BA. Opioids in arthroplasty: mind the gap between north america and the rest of the world. J Bone Joint Surg Am. 2018;100(24):2162–71. Available from: https://pubmed.ncbi.nlm.nih.gov/30562297/10.2106/JBJS.17.01422 30562297

[6] Anger M, Valovska T, Beloeil H, Lirk P, Joshi GP, Van de Velde M et al. PROSPECT guideline for total hip arthroplasty: a systematic review and procedure-specific postoperative pain management recommendations. Anaesthesia. 2021;76(8):1082–97. Available from: https://pubmed.ncbi.nlm.nih.gov/34015859/10.1111/anae.15498 34015859

[7] Girón-Arango L, Peng PWH, Chin KJ, Brull R, Perlas A. Pericapsular nerve group (PENG) block for hip fracture. Reg Anesth Pain Med. 2018;43(8):859–63. Available from: https://pubmed.ncbi.nlm.nih.gov/30063657/10.1097/AAP.0000000000000847 30063657

[8] Zheng J, Pan D, Zheng B, Ruan X. Preoperative pericapsular nerve group (PENG) block for total hip arthroplasty: a randomized, placebo-controlled trial. Reg Anesth Pain Med. 2022;47(3):155–60. 34873023 10.1136/rapm-2021-103228

[9] Chung CJ, Eom DW, Lee TY, Park SY. Reduced opioid consumption with pericapsular nerve group block for hip surgery: a randomized, double-blind, placebo-controlled trial. Pain Res Manag. 2022;2022(1):6022380. 10.1155/2022/6022380 PMC9780006 36569462

[10] Lin DY, Brown B, Morrison C, Fraser NS, Chooi CSL, Cehic MG et al. The pericapsular nerve group (PENG) block combined with Local infiltration analgesia (LIA) compared to placebo and LIA in hip arthroplasty surgery: a multi-center double-blinded randomized-controlled trial. BMC Anesthesiol. 2022;22(1):1–9. Available from: 10.1186/s12871-022-01787-2 10.1186/s12871-022-01787-2 PMC9356515 35933328

[11] Short AJ, Barnett JJG, Gofeld M, Baig E, Lam K, Agur AMR, et al. Anatomic study of innervation of the anterior hip capsule: implication for Image-Guided intervention. Reg Anesth Pain Med. 2018;43(2):186–92. 29140962 10.1097/AAP.0000000000000701

[12] Kitcharanant N, Leurcharusmee P, Wangtapun P, Kantakam P, Maikong N, Mahakkanukrauh P et al. Surgeon-performed pericapsular nerve group (PENG) block for total hip arthroplasty using the direct anterior approach: a cadaveric study. Reg Anesth Pain Med. 2022;47(6):359–63. 10.1136/rapm-2022-103482 35288453

[13] Yamak Altinpulluk E, Galluccio F, Salazar C, Espinoza K, Olea MS, Hochberg U et al. Peng block in prosthetic hip replacement: a cadaveric radiological evaluation. J Clin Anesth. 2020;65:109888–109888. 10.1016/j.jclinane.2020.109888 32447169

[14] Light TR, Keggi KJ. Anterior approach to hip arthroplasty. Clin Orthop Relat Res. 1980;152:255–60. Available from: https://www.researchgate.net/publication/15759712_Anterior_Approach_to_Hip_Arthroplasty 7438611

[15] Faldini C, Rossomando V, Brunello M, D’Agostino C, Ruta F, Pilla F et al. Anterior minimally invasive approach (AMIS) for total hip arthroplasty: analysis of the first 1000 consecutive patients operated at a high volume center. J Clin Med. 2024;13(9):2617. Available from: https://pubmed.ncbi.nlm.nih.gov/38731146/10.3390/jcm13092617 PMC11084447 38731146

[16] Hip Knee Spine Navigation Surgical Technique Surgical Technique TM Thanks to A C K N O W. L E D G M E N T S.

[17] Wylde V, Rooker J, Halliday L, Blom A. Acute postoperative pain at rest after hip and knee arthroplasty: severity, sensory qualities and impact on sleep. Orthop Traumatol: Surg Res. 2011;97(2):139–44. Available from: 10.1016/j.otsr.2010.12.003 10.1016/j.otsr.2010.12.003 21388906

[18] Pires Sousa I, Leite da Silva Peixoto CI, Fernandes Coimbra LA, da Costa Rodrigues FM. Comparison of pericapsular nerve group (PENG) block and epidural analgesia following total hip arthroplasty: a retrospective analysis. Revista Española de Anestesiología y Reanimación (English Edition). 2022;69(10):632–9. Available from: https://linkinghub.elsevier.com/retrieve/pii/S2341192922001822 10.1016/j.redare.2022.10.002 36376187

[19] Di Pietro S, Maffeis R, Girón-Arango L, Jannelli E, Perlini S. Pericapsular nerve group block for intracapsular vs. extracapsular hip fracture. Anaesthesia. 2025;81(1):140. Disponible en: 10.1111/anae.70030 10.1111/anae.70030 PMC12747586 41078268

[20] Di Pietro S, Maffeis R, Jannelli E, Mascia B, Resta F, De Silvestri A, Comparing the pericapsular nerve group block and fascia iliaca block for acute pain management in patients with hip fracture: a randomised clinical trial. Anaesthesia. 2025;80(12):1484–1492 10.1111/anae.16695 PMC12614414 40727959

[21] Petrovic NM, Milovanovic DR, Ignjatovic Ristic D, Riznic N, Ristic B, Stepanovic Z et al. Factors associated with severe postoperative pain in patients with total hip arthroplasty. Acta Orthop Traumatol Turc. 2014;48(6):615–22. 10.3944/AOTT.2014.14.0177 25637724

[22] Lunn TH, Husted H, Solgaard S, Kristensen BB, Otte KS, Kjersgaard AG, et al. Intraoperative local infiltration analgesia for early analgesia after total hip arthroplasty: a randomized, double-blind, placebo-controlled trial. Reg Anesth Pain Med. 2011;36(5):424–9. 21610559 10.1097/AAP.0b013e3182186866

[23] Martinez V, Belbachir A, Jaber A, Cherif K, Jamal A, Ozier Y, et al. The influence of timing of administration on the analgesic efficacy of parecoxib in orthopedic surgery. Anesth Analg. 2007;104(6):1521–7. 17513652 10.1213/01.ane.0000262039.69513.9d PMC2564988

[24] Pandazi A, Kanellopoulos I, Kalimeris K, Batistaki C, Nikolakopoulos N, Matsota P, et al. Periarticular infiltration for pain relief after total hip arthroplasty: a comparison with epidural and PCA analgesia. Arch Orthop Trauma Surg. 2013;133(11):1607–12. 24036613 10.1007/s00402-013-1849-8

[25] Ventafridda V, Saita L, Ripamonti C, De Conno F. WHO guidelines for the use of analgesics in cancer pain. Int J Tissue React. 1985;7(1):93–6. Available from: http://www.ncbi.nlm.nih.gov/pubmed/2409039 2409039

[26] Pascarella G, Costa F, Del Buono R, Pulitanò R, Strumia A, Piliego C et al. Impact of the pericapsular nerve group (PENG) block on postoperative analgesia and functional recovery following total hip arthroplasty: a randomised, observer-masked, controlled trial. Anaesthesia. 2021;76(11):1492–8. Available from: https://pubmed.ncbi.nlm.nih.gov/34196965/10.1111/anae.15536 PMC8519088 34196965

[27] Huda AU, Ghafoor H. The use of pericapsular nerve group (PENG) block in hip surgeries is associated with a reduction in opioid consumption, less motor block, and better patient satisfaction: a meta-analysis. Cureus. 2022;14(9):e28872. 10.7759/cureus.28872 PMC9449447 36105907

[28] Kinder KD, Stambough JB, Barnes CL, Porter A, Mears SC, Stronach BM. Pericapsular nerve group block did not reduce postoperative pain or opioid use after total hip arthroplasty. J Arthroplasty. 2024;39(9):S112–6. 39019412 10.1016/j.arth.2024.06.043 PMC11339861

[29] Yang Xteng, Huang H feng, Sun L, Yang Z, Deng C, yong. Tian X bin. Direct anterior approach versus posterolateral approach in total hip arthroplasty: a systematic review and meta-analysis of randomized controlled studies. Orthop Surg. 2020;12(4):1065–73. 10.1111/os.12669 PMC7454221 32558261

[30] Behrends M, Yap EN, Zhang AL, Kolodzie K, Kinjo S, Harbell MW, et al. Preoperative fascia Iliaca block does not improve analgesia after arthroscopic hip surgery, but causes quadriceps muscles weakness: a randomized, double-blind trial. Anesthesiology. 2018;129(3):536–43. 29975203 10.1097/ALN.0000000000002321

[31] Pascarella G, Costa F, Del Buono R, Pulitanò R, Strumia A, Piliego C, et al. Impact of the pericapsular nerve group (PENG) block on postoperative analgesia and functional recovery following total hip arthroplasty: a randomised, observer-masked, controlled trial. Anaesthesia. 2021;76(11):1492–8. 34196965 10.1111/anae.15536 PMC8519088

[32] Nájera Losada DC, Pérez Moreno JC. Pericapsular nerve group block in hip surgery. An alternative that goes beyond what we know? Rev Esp Anestesiol Reanim. 2022;69(10):654–62. Available from: https://pubmed.ncbi.nlm.nih.gov/36344408/10.1016/j.redare.2021.10.002 36344408

[33] Martínez Martín A, Pérez Herrero M, Sánchez Quirós B, López Herrero R, Ruiz Bueno P, Cocho Crespo S. Benefits of analgesic blocks, PENG block (PEricapsular Nerve Group), in fast recovery after hip surgery. Rev Esp Cir Ortop Traumatol. 2023;67(1):27–34. Available from: https://pubmed.ncbi.nlm.nih.gov/35483667/10.1016/j.recot.2022.10.005 36243392

[34] The IDEAL Framework. Ideal. 2025 [citado el 22 de diciembre de 2025]. Disponible en: https://www.ideal-collaboration.net/the-ideal-framework-2/

